# Vinpocetine Overcomes Paclitaxel Resistance in a Triple‐Negative Breast Cancer Cell Line

**DOI:** 10.1002/cbin.70088

**Published:** 2025-09-30

**Authors:** Hsiao‐Hui Kuo, Chien‐Wei Huang, Wei‐Rou Chiang, Ling‐Huei Yih

**Affiliations:** ^1^ Institute of Cellular and Organismic Biology Academia Sinica Taipei Taiwan

**Keywords:** cell death, P‐glycoprotein, Paclitaxel, phosphodiesterase 1, Vinpocetine

## Abstract

Paclitaxel is a first‐line treatment for triple‐negative breast cancer (TNBC), but its efficacy is commonly limited by tumor‐cell resistance. Vinpocetine is a well‐tolerated dietary supplement with pleiotropic cellular effects, including potential anti‐tumor activity. In this study, we tested whether and how vinpocetine might enhance the sensitivity of TNBC cells to paclitaxel. A paclitaxel‐resistant TNBC cell line (T50RN) was established by incubating MDA‐MB‐231 cells with escalating concentrations of paclitaxel (0.5–50 nM). The effects of vinpocetine on T50RN cell sensitivity to paclitaxel were examined. T50RN cells were significantly more resistant to paclitaxel than the parental MDA‐MB‐231 cells. Vinpocetine itself was slightly cytotoxic to cells but considerably enhanced paclitaxel sensitivity in T50RN cells. Expression of PDE1C, a target of vinpocetine, was elevated in T50RN cells. Depletion of PDE1C moderately enhanced paclitaxel sensitivity of T50RN cells, suggesting that PDE1C overexpression might contribute to paclitaxel resistance. In addition, vinpocetine induced microtubule stabilization and significantly enhanced paclitaxel‐induced microtubule stabilization. However, vinpocetine could still enhance paclitaxel sensitivity in PDE1C‐depleted T50RN cells, indicating that vinpocetine also acts through factor(s) other than PDE1C. P‐gp expression and activity were elevated in T50RN cells, and inhibition of P‐gp sensitized T50RN cells to paclitaxel. Vinpocetine functionally disrupted P‐gp in T50RN cells and further enhanced the death of P‐gp‐inhibited paclitaxel‐treated T50RN cells. Thus, our results revealed that vinpocetine may act on P‐gp and PDE1C to facilitate paclitaxel accumulation and paclitaxel‐induced stabilization of microtubules in T50RN cells, thereby enhancing the antimitotic effects of paclitaxel and disrupting paclitaxel‐resistance mechanisms.

**Abbreviations**
ABCB1: ATP binding cassette subfamily B member 1
IKK/NF‐κB: I kappa B kinase/nuclear factor kappa B
Nrf2: nuclear factor erythroid 2‐related factor 2
PDE1: phosphodiesterase 1
P‐gp/MDR1: P‐glycoprotein/Multidrug resistance 1
ROS: reactive oxygen species
STAT3: signal transducer and activator of transcription factor 3
TMRE: tetramethylrhodamine ethyl ester
TNBC: triple‐negative breast cancer.

## 1 Introduction

Paclitaxel is a first‐line anticancer drug that binds tubulin directly and with high affinity. Binding of paclitaxel along the length of microtubules promotes their polymerization and stabilization in living cells, resulting in the disruption of mitotic spindle assembly, mitotic arrest, and cell death (Jordan and Wilson 2004). The ability of paclitaxel to induce catastrophic multipolar mitosis and chromosome missegregation is fundamental to its therapeutic effect in cancer treatment (Weaver 2014). However, resistance and toxic side effects often develop in patients receiving paclitaxel‐based treatments, limiting the drug efficacy. Resistance to paclitaxel can be developed through a variety of mechanisms, including increased drug efflux, upregulation of cell survival pathways, evasion of apoptosis, altered tubulin expression, and metabolic reprogramming (Das et al. 2021; Murray et al. 2012). These resistance mechanisms not only lead to treatment failure but may also further drive cancer progression and disease recurrence, accelerating poor outcomes for patients. Thus, an urgent clinical need exists for drugs that can augment paclitaxel‐induced antimitotic effects and overcome paclitaxel resistance mechanisms.

Vinpocetine is a pleiotropic semisynthetic derivative of the vinca alkaloid vincamine, which is extracted from the periwinkle plant. This molecule can pass the blood–brain‐barrier (Bönöczk et al. 2000; Lin et al. 2014), has an excellent safety profile, and has been used as a dietary supplement to prevent or treat stroke, cerebrovascular disorders, senile dementia and memory disturbances (Ren et al. 2023; Y. S. Zhang et al. 2018). Several different mechanisms of action have been reported for vinpocetine. For instance, its therapeutic activity in neurological disease may be attributed to modulatory effects on phosphodiesterase 1 (PDE1), voltage‐gated sodium channels, and I kappa B kinase/nuclear factor kappa B (IKK/NF‐κB) (Jeon et al. 2010). In addition, vinpocetine has been shown to target multiple mitochondrial proteins (Svab et al. 2019). Since treatment with vinpocetine was reported to improve cognitive impairment in mice and nasopharyngeal carcinoma patients with radiation‐induced brain injuries (Hu et al. 2022; P. Zhang et al. 2021), it may be useful as a supportive therapy for cancer patients. Moreover, laboratory studies have shown that vinpocetine inhibits breast cancer cell growth, possibly by attenuating signaling through Akt and Signal transducer and activator of transcription factor (STAT)‐3 (Huang et al. 2012). The molecule also enhances cisplatin sensitivity of non‐small cell lung cancer cells by reducing Nuclear factor erythroid 2‐related factor 2 (Nrf2) signaling (Zhuang et al. 2022). Finally, vinpocetine was shown to improve tumor oxygenation and potentiate radiation effects in murine models of squamous cell cancer (Amano et al. 2005). These multifaceted actions of vinpocetine, including vasodilation, oxidation and anti‐inflammation effects, may provide beneficial effects in cancer patients receiving therapy. Therefore, vinpocetine is an intriguing candidate for use in combination with paclitaxel.

Breast cancer is one of the most common cancers in women worldwide. In particular, triple‐negative breast cancer (TNBC) accounts for about 10%–15% of all breast cancers and is associated with a poor prognosis, including high recurrence rates and distant metastasis (Tan and Dent 2018). Paclitaxel is one of the first‐line therapies given to TNBC patients. However, many patients experience treatment failure with tumor recurrence and metastatic disease (Foulkes et al. 2010), which is often associated with developed resistance to paclitaxel. For laboratory studies on paclitaxel resistance, the MDA‐MB‐231 cell line is a useful in vitro model. In this TNBC cell line, approximately half of the cell population carries extra centrosomes (Kwon et al. 2008), which tend to cluster into pseudo‐bipolar spindles during mitosis to facilitate cell division and survival (Kwon 2016). This mechanism can allow the cells to evade toxicity from anti‐mitosis drugs (Sinnott et al. 2014). In this study, we established a paclitaxel‐resistant cell line, T50RN, from the MDA‐MB‐231 cell line and investigated whether and how vinpocetine could enhance paclitaxel sensitivity and overcome paclitaxel resistance. Our results show that vinpocetine may act by disrupting P‐glycoprotein/Multidrug resistance 1 (P‐gp/MDR1) and PDE1 activities to enhance paclitaxel accumulation and paclitaxel‐induced stabilization of microtubules, thereby increasing the anti‐mitotic effect and cytotoxicity of paclitaxel in T50RN cells.

## 2 Materials and Methods

### 2.1 Cell Culture and Chemicals

MDA‐MB‐231 cells were purchased from American Type Culture Collection and maintained as previously described (Fang et al. 2021). A paclitaxel‐resistant cell line, T50RN, was established according to a previous protocol (Fang et al. 2021). Briefly, MDA‐MB‐231 cells were exposed to stepwise escalating concentrations (0.5–50 nM) of paclitaxel (Paclitaxel, Merck, Darmstadt, Germany). Resistant cells (T50RN) were isolated and maintained in medium containing 50 nM paclitaxel. Small molecules used in this study included rhodamine 123 and tiron (Sigma, Saint Louis, MO, USA), vinpocetine and zosuquidar (Zo) (Cayman Chemical, Ann Arbor, MI, USA).

### 2.2 Cytotoxicity

Cytotoxicity was assessed with the trypan blue exclusion assay for cell viability (Fang et al. 2021) or the colony formation assay (Fang et al. 2021), as previously described. For the trypan blue exclusion assay, logarithmically growing cells on plates were treated for 72 h. After the treatment, cells were collected and stained with trypan blue (0.4% w/v). The transparent viable cells and blue dead cells were counted on a hemocytometer. For the colony formation assay, logarithmically growing cells were treated for 24 h and then replated at low density for 10 days. During this period, single cells grew into colonies, which were fixed with methanol, stained with crystal violet (0.5% w/v) and counted using an inverted microscope. The resistance index (RI) was calculated by the ratio of the IC_50_ of resistant cell line to wild‐type cell line (Sazonova et al. 2024). The cytotoxicity of the combination treatment was determined using the combination index (CI) calculated by CompuSyn software (version 1.0.1; CompuSyn, Paramus, NJ) according to the theory of Chou–Talalay (Chou 2010).

### 2.3 Immunofluorescence Staining and Analysis of Mitotic Spindles Abnormalities

Cells seeded on glass coverslips were treated and fixed. Then, immunofluorescence staining of mitotic spindles was performed as previously described (Fang et al. 2016). Primary antibodies included anti‐α‐tubulin (T5168, Sigma or GTX112141, GeneTex) and anti‐γ‐tubulin (T6557 or T3559, Sigma). Secondary antibodies, Alexa‐Fluor 488‐ or 633‐conjugated goat anti‐mouse or anti‐rabbit IgG, were purchased from Invitrogen (Carlsbad, CA, USA). Images of the immunostained samples were obtained with a confocal microscope (Zeiss LSM 980, Oberkochen, Germany). The numbers of cells with indicated spindle types were counted using a Zeiss Axioplan 2 Imaging MOT fluorescence microscope.

### 2.4 Analysis of Cell Cycle Progression and Mitotic Index

Cell cycle progression was monitored using DNA flow cytometry, as previously described (Fang et al. 2021). The percentage of cells in mitosis was measured by flow cytometry analysis of phospho‐histone H3 (p‐H3, the mitosis marker)‐positive cells.

### 2.5 Detection of Apoptosis

Apoptosis was detected with an annexin V‐FITC/PI staining kit (BD Bioscience, New Jersey, USA) as described previously (Yih, Hsueh, Luu, Chiu, & Lee 2005). Briefly, cells were collected after treatment for 72 h. The cells were rinsed, resuspended at 5 × 10^5^ cells per 400 μL binding buffer, and incubated with 2 μL annexin V‐FITC and propidium iodide for 15 min in the dark at room temperature. Afterward, the cells were analyzed by flow cytometry (Attune NxT, Thermo Fisher Scientific).

### 2.6 Q‐PCR

Total RNA was extracted from cells with Trizol (Thermo Fisher Scientific) following the manufacturer's protocol. Then, 2.5 μg of total RNA was reverse‐transcribed into cDNA with the PrimeScriptII RT Enzyme kit (Takara Bio USA). qPCR was performed with a Light Cycler 480 system (Roche, Penzberg, Germany). The amplification reaction was a 20 μL mixture containing 10 μL 2× SYBR Green mix, 1 μL cDNA, and 0.2 μM of each primer. The PCR conditions were as follows: denaturation at 95°C for 5 min; 40 cycles of 95°C for 10 s, 60°C for 10 s, and 72°C for 10 s. Following amplification, the PCR products were examined via melting curve analyses. ATP binding cassette subfamily B member 1 (ABCB1) and PDE1C mRNA levels were normalized to GAPDH mRNA levels in the same sample; each sample was analyzed in triplicate. mRNA levels were assessed by calculating 2^−ΔΔCt^ values. The primer sets were as follows: ABCB1, forward 5′‐GAGGCTCTATGACCCCAC‐3′ and reverse 5′‐CACACCAATGATTTCCCGT‐3′; PDE1C, forward 5′‐CTTGTCTCATTTGTGGAGGC‐3′ and reverse 5′‐CACTGTCTGTGTAACATCGG‐3′; GAPDH, forward 5′‐CTCCTCCACCTTTGACG‐3′ and reverse 5′‐ACCACCCTGTTGCTGTA‐3′.

### 2.7 Depletion of Cellular PDE1C

Depletion of PDE1C was achieved by transducing T50RN cells with VSV‐G‐pseudotyped lentivirus‐based short hairpin RNA (shRNA), as previously described (Fang et al. 2021). The shRNAs targeting PDE1C (TRCN48759 and TRCN48760) were purchased from the National RNAi Core Facility (Genomic Research Center, Academia Sinica). Cells were transduced with pLKO.1‐ or shRNA‐containing supernatant in growth medium supplemented with 10 μg/mL polybrene. At 24 h post‐transduction, 2 μg/mL puromycin was added to culture medium to select for stable clones. Alternatively, cells were subjected to paclitaxel treatment after transduction for at least 2 days. The depletion efficiency was verified by immunoblotting.

### 2.8 Detection of Intracellular Ca^2+^

Intracellular Ca^2+^ was measured by Fluo‐8 staining. Cells were preloaded with Fluo‐8 (2 μM) for 50 min, followed by vinpocetine or paclitaxel treatment for 20 min. Flou‐8 intensities were measured by flow cytometry (Attune NxT, Thermo Fisher Scientific). Alternatively, the cells were treated with vinpocetine or paclitaxel for 24 h, then loaded with Fluo‐8 (2 μM) for 50 min before intracellular intensities of Fluo‐8 were measured by flow cytometry (Attune NxT, Thermo Fisher Scientific).

### 2.9 Measurement of Cyclic Adenosine Monophosphate (cAMP)

Intracellular cAMP levels were measured using the cAMP Competitive ELISA kit (Cell Signaling Technology) according to the manufacturer's protocol. Briefly, T50RN cells were untreated or treated for 24 h. Then, 3 × 10^6^ cells were lysed with cell lysis buffer (provided in the kit). Free cAMP in cell lysates and a fixed amounts of HRP‐labeled cAMP were loaded onto wells coated with anti‐cAMP antibodies. The cAMP in cell lysates competed with the fixed amount of HRP‐linked cAMP for binding to anti‐cAMP antibodies on the plate. After a wash to remove excess sample cAMP and HRP‐linked cAMP, the amount of bound HRP‐labeled cAMP was measured using a fluorometric HRP substrates. The magnitude of signal was inversely proportional to the quantity of cAMP in the sample. The level of signal was quantified by comparison to a cAMP standard curve, which was run at the same time as the tested samples.

### 2.10 Quantification of Microtubule Polymerization

Microtubule polymerization was measured in terms of microtubule polymer mass, using the “Tubeness” plugin in ImageJ software as previously described (Harkcom et al. 2014). Polymerized tubulin (revealed by immunofluorescence staining of α‐tubulin) was first identified and quantified using the “Tubeness” plugin on a 3D image stack. The total cellular area was determined by the “Contour” plugin. The ratio of polymerized tubulin mass to cellular area was taken as a measure of microtubule polymerization.

### 2.11 Immunoblotting

Cell lysis and immunoblotting were carried out as described (Fang et al. 2016). Specific proteins were detected using antibodies against PDE1C (Invitrogen), acetylated tubulin (Cell Signaling Technology, Danvers, MA), α‐tubulin (GeneTex), and P‐gp/MDR1 (Cell Signaling Technology). GAPDH or PCNA loading controls were respectively detected with anti‐GAPDH (Genetex) or anti‐PCNA (Santa Cruz Biotechnology Inc., Dallas, Texas).

### 2.12 Analysis of Reactive Oxygen Species (ROS) and Mitochondrial Membrane Potential

ROS generation was measured with CellROX Green or MitoSox Red Reagents (Thermo Fisher Scientific). After treating T50RN cells with vinpocetine for 6 h, the cells were collected and stained with 0.5 μM CellROX Green or 2 μM MitoSox Red for 30 min at 37°C. After thorough rinsing, the cells were analyzed with flow cytometry (Attune NxT, Thermo Fisher Scientific). The fluorescent dye tetramethylrhodamine ethyl ester (TMRE) was used to measure mitochondrial membrane potential. In brief, treated T50RN cells were collected and incubated in medium containing 50 nM TMRE for 5 min in the dark at room temperature. After thorough rinsing with PBS, the cells were immediately analyzed with a flow cytometer.

### 2.13 Rhodamine 123 Accumulation and Efflux Assays

Cellular rhodamine 123 accumulation and efflux were measured as indicators of P‐gp activity (Pétriz and García‐López 1997). For the rhodamine 123 accumulation assay, cells were untreated or treated with vinpocetine or Zosuquidar for 24 h. Then cells were incubated with rhodamine 123 (2 μM) for another 1 h. Cells were then collected, and the intensity of cellular rhodamine 123 was analyzed by flow cytometry (Attune NxT, Thermo Fisher Scientific). For the rhodamine 123 efflux assay, cells were first loaded with 2 μM rhodamine 123 for 1 h. Then, the dye was washed out, and the cells were incubated in rhodamine‐free medium for another 1–2 h with or without vinpocetine. At the indicated time, cells were collected, and rhodamine 123 retention was assessed by flow cytometry (Attune NxT, Thermo Fisher Scientific).

### 2.14 Statistical Analysis

All experiments were performed at least three times. The results are shown as mean ± standard deviation (SD). The treated cell populations were compared with vehicle controls in each experiment. The differences between groups were analyzed by Student's *t*‐test or by two‐way analysis of variance (two‐way ANOVA) and Tukey's multiple comparison test using GraphPad Prism 10.1. Statistical significance was set as *p* < 0.05.

## 3 Results

### 3.1 Paclitaxel Resistance of T50RN Cells

T50RN cells were established from MDA‐MB‐231 cells by culturing the parental cells in stepwise escalating paclitaxel concentrations (0.5–50 nM). The paclitaxel‐exposed MDA‐MB‐231 cells were grown to sub‐confluency and sub‐cultured for the next round of paclitaxel exposure, until the paclitaxel concentration reached 50 nM. A resistant T50RN cell line was obtained, expanded and maintained in culture medium containing 50 nM paclitaxel.

**Figure 1:**
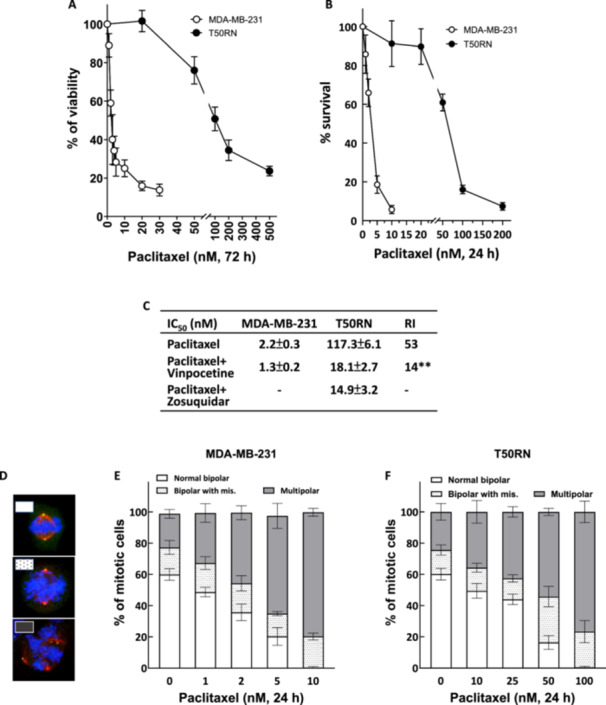
T50RN cells are more resistant to paclitaxel than parental MDA‐MB‐231 cells. (A and B) Cytotoxicity of paclitaxel in MDA‐MB‐231 and T50RN cells. Cells were treated for 72 h (A) or 24 h (B) with paclitaxel at the indicated concentrations and then subjected to the trypan blue exclusion assay (A) or colony forming assay (B). The relative cell viabilities or survival compared to vehicle control are shown as mean ± SD from at least three independent experiments. (C) IC_50_ and resistance index (RI). MDA‐MB‐231 or T50RN cells were treated as in Figures 1A, 2B,C, and 3F, then the IC_50_ was calculated according to Prism 10.1. The RI was calculated by the ratio of the IC_50_ of T50RN to MDA‐MB‐231 in the same treatment. (D) Representative images of untreated MDA‐MB‐231 cells with (upper) normal mitotic spindle, (middle) bipolar‐like spindle with misaligned chromosomes (arrow head), and (bottom) paclitaxel‐treated cells with multipolar spindles. Cells were fixed and immunostained for γ‐tubulin (green), α‐tubulin (red) and chromosomes (blue). (E and F) Paclitaxel dose‐dependently induced spindle abnormalities in MDA‐MB‐231 (E) and T50RN (F) cells. Cells were treated for 24 h with the indicated concentrations of paclitaxel, followed by analysis of mitotic spindles. Percentages of mitotic cells with the indicated spindle types are shown as mean ± SD from at least three independent experiments.

To evaluate the level of paclitaxel resistance in T50RN cells, we first conducted a trypan blue exclusion assay. Treatment of cells with paclitaxel for 72 h dose‐dependently reduced viability of the parental MDA‐MB‐231 cells (Figure 1A), yielding an IC_50_ of 2.2 nM (calculated with Prism 10.1). In contrast, T50RN cells were significantly more resistant to paclitaxel treatment, according to the trypan blue exclusion assay (IC_50_ 117.3 nM; Figure 1A). Paclitaxel resistance of T50RN cells was also assessed with the colony formation assay. The results showed that T50RN was considerably more resistant to paclitaxel than its parental MDA‐MB‐231 cell line (Figure 1B). Figure 1C showed that the RI to paclitaxel, as determined by the ratio of the IC_50_ of resistant T50RN to parental MDA‐MB‐231, was 53. Next, we examined paclitaxel‐induced spindle abnormalities. In the untreated MDA‐MB‐231 cultures, about 60% of mitotic cells displayed normal bipolar spindles (Figure 1D [upper]), 20% showed a bipolar‐like structure but with misaligned or lagged chromosomes (Figure 1D [middle]), and the remaining 20% had multipolar spindles (Figure 1D [bottom] and 1E). These observations are consistent with previous reports (Kwon 2016). Treatment of MDA‐MB‐231 cells with 1–10 nM paclitaxel for 24 h dose‐dependently increased the percentage of mitotic cells with multipolar spindles (Figure 1E, gray bar) and decreased the proportion of cells with normal bipolar spindles (Figure 1E, white bar). Untreated T50RN cells showed a similar distribution pattern for mitotic spindles as the MDA‐MB‐231 cells. Paclitaxel treatment induced multipolar spindles in T50RN cells at concentrations higher than 10 nM (Figure 1F, gray bar), and much higher paclitaxel concentrations were required to induce levels of spindle defects similar to those seen in parental cells. Based on these observations, we concluded that T50RN cells are more resistant to paclitaxel than MDA‐MB‐231 cells in terms of spindle abnormalities and cell death.

**Figure 2:**
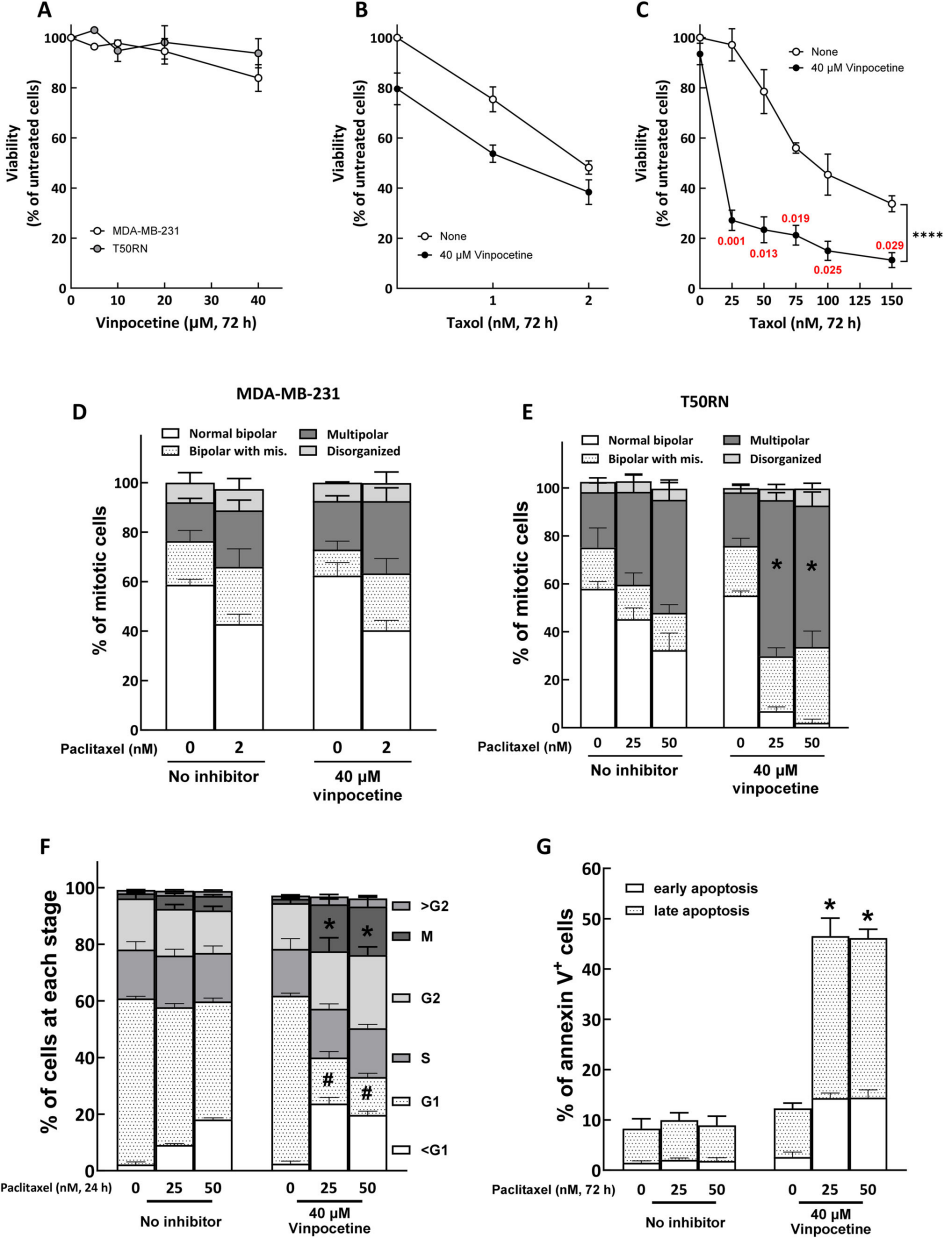
Vinpocetine enhances T50RN sensitivity to paclitaxel. (A) Cytotoxicity of vinpocetine in MDA‐MB‐231 and T50RN cells. Cells were treated with vinpocetine for 72 h at the indicated concentrations and then subjected to the trypan blue exclusion assay. (B and C) Vinpocetine enhanced sensitivity to paclitaxel in T50RN cells but not in MDA‐MB‐231 cells. MDA‐MB‐231 (B) or T50RN (C) cells were treated with paclitaxel alone or paclitaxel plus vinpocetine as indicated for 72 h and then subjected to trypan blue exclusion assay. The relative cell viabilities compared to vehicle control are shown as mean ± SD from at least three independent experiments. Significant differences between treatment groups were assessed by two‐way ANOVA. Red numbers in panel C indicate CI, where CI = 1 indicates that the two drugs have additive effects, CI < 1 indicates synergism, and CI > 1 indicates antagonism. (D and E) Vinpocetine enhanced paclitaxel‐induced spindle abnormalities in T50RN cells but not in MDA‐MB‐231 cells. MDA‐MB‐231 (D) or T50RN (E) cells were treated with paclitaxel alone or paclitaxel plus vinpocetine as indicated for 24 h and then subjected to analysis of mitotic spindles. Percentages of mitotic cells with the indicated spindle types are shown as mean ± SD from at least three independent experiments. *p < 0.05 comparing the percentages of mitotic cells with multipolar spindles in paclitaxel plus vinpocetine‐treated to those in corresponding paclitaxel alone‐treated T50RN cells; Student's *t* test. (F) Vinpocetine enhanced paclitaxel‐induced mitotic arrest in T50RN cells. T50RN cells were treated with paclitaxel alone or paclitaxel plus vinpocetine as indicated for 24 h and then were fixed, stained for phospho‐histone H3 and DNA content, and analyzed by flow cytometry. The mean ± SD from three independent experiments is shown. # and *p < 0.05 comparing the percentages of G1 (#) and mitotic (*) cells in paclitaxel plus vinpocetine‐treated to those in corresponding paclitaxel alone‐treated T50RN cells; Student's *t* test. (G) Vinpocetine enhanced paclitaxel‐induced apoptosis in T50RN cells. T50RN cells were treated with paclitaxel alone or paclitaxel plus vinpocetine as indicated for 72 h and then subjected to flow cytometry analysis of annexin V‐positive cells. The mean ± SD from three independent experiments is shown. *p < 0.05 comparing the percentages of total apoptotic cells in paclitaxel plus vinpocetine‐treated to those in corresponding paclitaxel alone‐treated T50RN cells; Student's *t* test.

**Figure 3:**
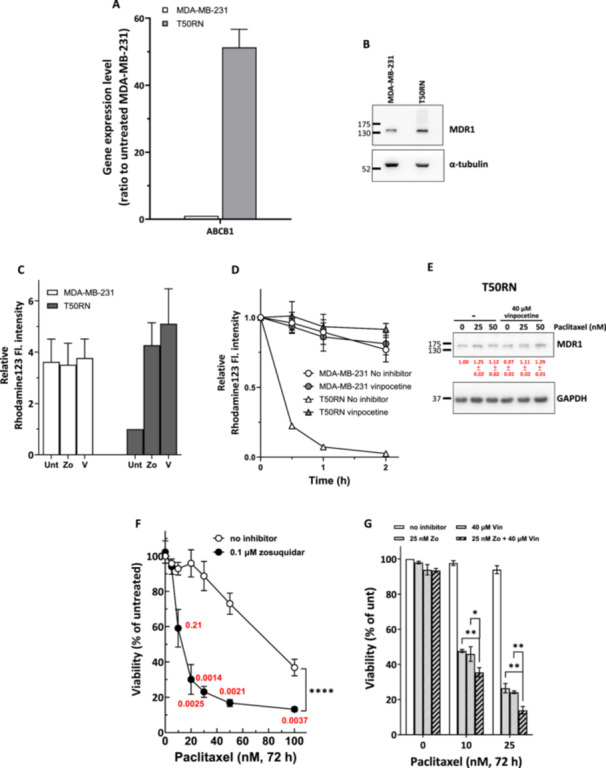
*ABCB1*/P‐gp expression is elevated in T50RN cells and inhibition of P‐gp sensitizes T50RN cells to paclitaxel. The expression levels of *ABCB1* mRNA (A) and P‐gp/MDR1 protein (B) were significantly elevated in T50RN cells compared to MDA‐MB‐231 cells. (C) Rhodamine 123 accumulation was much less in T50RN cells than in MDA‐MD‐231 cells. Accumulation could be rescued by treatment with Zosuquidar or vinpocetine. Cells were untreated or treated with 0.1 µM Zosuquidar (Zo) or 40 µM vinpocetine (V) for 24 h, then loaded with rhodamine 123 for another 1 h, and analyzed by flow cytometry. (D) Rhodamine 123 efflux was much greater in T50RN cells than in MDA‐MB‐231 cells. Efflux could be inhibited by vinpocetine. Cells were first loaded with rhodamine 123 for 1 h and incubated in rhodamine 123‐free medium. During the incubation, cells were either left untreated or treated with 40 µM vinpocetine for the indicated times and analyzed by flow cytometry. (E) Vinpocetine treatment had no significant effect on P‐gp/MDR1 protein expression. T50RN cells were treated as indicated and then collected for immunoblotting analysis of P‐gp/MDR1 protein level. GAPDH is shown as a loading control. Numbers (means ± SD) indicate the quantification of protein bands from three independent experiments. The intensity of each protein band measured using GeneTools (version 4.3) from Syngene was normalized to the intensity of the GAPDH band and then compared with that of the untreated group. (F) Zosuquidar significantly enhanced paclitaxel sensitivity in T50RN cells. Red numbers indicate CI, where CI < 1 indicates synergism. (G) Vinpocetine had no measurable effect on Zosuquidar/paclitaxel‐cotreated T50RN cells. T50RN cells were treated as indicated for 72 h and subjected to trypan blue exclusion analysis. The relative cell viabilities compared to vehicle control are shown as mean ± SD from at least three independent experiments. Significant differences in the data were assessed by two‐way ANOVA. ****p < 0.0001 comparing between indicated groups.

### 3.2 Vinpocetine Enhances Paclitaxel Cytotoxicity in T50RN

We next tested whether vinpocetine could modulate paclitaxel resistance of T50RN cells. Vinpocetine treatment alone (up to 40 μM) caused minor cytotoxicity in both MDA‐MB‐231 and T50RN cells (Figure 2A). Combined treatment of 40 μM vinpocetine and paclitaxel showed an additive cytotoxic effect in MDA‐MB‐231 cells (Figure 2B). However, the combination of paclitaxel and vinpocetine considerably enhanced T50RN sensitivity to paclitaxel, decreasing the IC_50_ of paclitaxel from ~100 nM to only 18.1 nM (Figure 2C). As shown in Figure 1C, the RI was significantly reduced from 53 to 14 comparing resistant to parental cells treated alone with paclitaxel and cotreated with paclitaxel and vinpocetine, indicating that vinpocetine was effective to enhancing paclitaxel cytotoxicity in T50RN cells. According to the CI theory of Chou–Talalay (Chou 2010), the CI (Figure 2C, red numbers) of our combined treatments are smaller than 1, suggesting that vinpocetine synergistically enhanced the cytotoxicity of paclitaxel in T50RN cells. Of note, vinpocetine alone (40 μM, 24 h) had no effects on the distributions of spindle patterns in either MDA‐MB‐231 or T50RN cells (Figure 2D,E). However, the frequency of paclitaxel‐induced spindle abnormalities was significantly elevated by cotreatment of T50RN cells with paclitaxel and vinpocetine (Figure 2E). No similar effect of the combination on spindle pattern was observed in MDA‐MB‐231 cells (Figure 2D).

The effects of vinpocetine on paclitaxel‐induced cell cycle arrest and apoptosis were also analyzed. The results of these experiments showed that vinpocetine could significantly enhance the population of paclitaxel‐induced sub‐G1 and mitotic T50RN cells at 24 h (Figure 2F) and apoptotic T50RN cells at 72 h (Figure 2G). These results suggest that vinpocetine enhancement of paclitaxel‐induced mitotic arrest and abnormal cell division might cause the cells to subsequently undergo apoptosis. In summary, our results to this point showed that vinpocetine can sensitize T50RN cells to paclitaxel and appears to overcome paclitaxel resistance.

### 3.3 Vinpocetine May Enhance T50RN Sensitivity to Paclitaxel Partially by Inhibiting PDE1C

**Figure 4:**
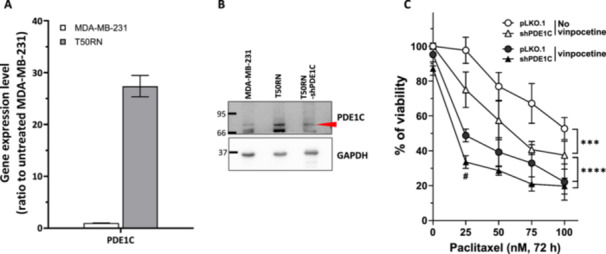
PDE1C expression is elevated in T50RN cells and depletion of PDE1C moderately enhances paclitaxel sensitivity of T50RN cells. The mRNA (A) and protein (B) expression levels of PDE1C were elevated in T50RN cells compared to MDA‐MB‐231 cells. (C) Depletion of PDE1C could moderately sensitize T50RN cells to paclitaxel. Logarithmically growing T50RN cells were transduced with *PDE1C*‐specific shRNAs or control pLKO.1 for 48 h. Then cells were treated with paclitaxel alone or paclitaxel plus vinpocetine as indicated for another 72 h. The relative cell viabilities compared to vehicle control are shown as mean ± SD from at least three independent experiments. Significant differences between treatment groups were assessed by two‐way ANOVA. ***p < 0.001, ****p < 0.0001 comparing between indicated groups.

Vinpocetine is known to function as a PDE1 inhibitor (Y. S. Zhang et al. 2018), so we examined the expression of PDE1 in T50RN cells. Our qPCR results showed that *PDE1C* (Figure 4A) expression was highly upregulated in T50RN cells as compared to MDA‐MB‐231 cells. Of note, expression levels of *PDE1A* and *PDE1B* were not different between the cell lines (data not shown). Depletion of PDE1C with specific shRNAs (Figure 4B) significantly increased T50RN cell sensitivity to paclitaxel as compared to pLKO.1‐transduced T50RN control cells (Figure 4C, unfilled circles vs. unfilled triangles). As such, the paclitaxel resistance of T50RN cells appears to be at least partially dependent on PDE1C. However, paclitaxel was more toxic to vinpocetine‐treated pLKO.1‐transduced T50RN cells than the PDE1C‐depleted T50RN cells (Figure 4C, solid circles vs. unfilled triangles), suggesting that vinpocetine might act through targets other than PDE1C. Moreover, vinpocetine could enhance paclitaxel sensitivity in PDE1C‐depleted T50RN cells (Figure 4C, solid triangles vs. unfilled triangles), further suggesting that vinpocetine acts through factor(s) in addition to PDE1C.

### 3.4 Vinpocetine Enhances Paclitaxel‐Induced Microtubule Polymer Assembly

**Figure 5:**
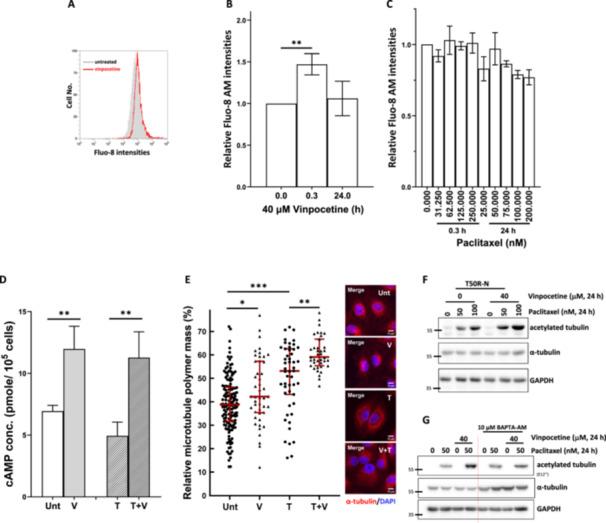
Vinpocetine enhances paclitaxel effects on microtubule polymer mass. (A) A representative histogram showing fluo‐8 distribution after vinpocetine treatment (red) in T50RN cells. (B) Vinpocetine increased intracellular calcium levels at 0.3 h in T50RN cells. T50RN cells were treated with vinpocetine as indicated, then stained with flou‐8, and analyzed by flow cytometry. (C) Paclitaxel did not significantly change calcium levels in T50RN cells. (D) Vinpocetine enhanced cAMP level in paclitaxel‐untreated or ‐treated T50RN cells. T50RN cells were untreated (Unt), treated with 40 µM vinpocetine (V), 25 nM paclitaxel (T), or their combination (T + V) for 24 h. Then the cells were subjected to cAMP analysis. **p < 0.01 comparing between indicated groups. (E and F) Vinpocetine stabilized microtubules and further enhanced paclitaxel‐induced microtubule stabilization. Quantification (E, left) and representative image (E, right) of microtubule polymer mass in T50RN cells, according to immunostaining for α‐tubulin. (G) cAMP concentration (*p*mol/10^5^ cells). The polymerized microtubules were identified using the “Tubeness” plugin in Fiji/ImageJ and normalized to cell area. Data show the distribution of percentages of polymerized microtubules in each cell with median ± interquartile range. Significant differences in the data were assessed by Mann–Whitney test. Unt, untreated cells; V, 40 µM vinpocetine; T, 25 nM paclitaxel; T + V, 25 nM paclitaxel plus 40 µM vinpocetine. *p < 0.05, **p < 0.01, ***p < 0.001 comparing between indicated groups. Microtubule polymerization was analyzed according to tubulin acetylation (E). T50RN cells were treated as indicated and then collected for immunoblotting analysis of acetylated tubulin and α‐tubulin. GAPDH is shown as a loading control.

Since PDE1 catalyzes the degradation of both cAMP and cGMP, its inhibition should increase cellular cAMP and cGMP levels. It has been shown that cAMP or cGMP can lead to Ca^2+^ influx (Bischof et al. 1995; Howe 2011), and intracellular Ca^2+^ is a critical modulator of microtubule dynamics (Babich and Burkhardt 2013; Means et al. 1982). Since our results showed that vinpocetine enhanced paclitaxel‐induced spindle abnormalities (Figure 2D), we next examined whether vinpocetine might affect intracellular Ca^2+^ levels and tubulin polymerization. Figure 5A,B shows that intracellular Ca^2+^ was transiently increased by treating T50RN cells with vinpocetine for 0.3 h and then returned to basal levels at 24 h. However, paclitaxel treatment had no detectable effect on intracellular Ca^2+^ levels (Figure 5C). In addition, vinpocetine also significantly increased cAMP level in T50RN cells either without (Figure 5D, V) or with (Figure 5D, T + V) paclitaxel treatment (Figure 5D). The level of cGMP was very low in T50RN cells and did not change after vinpocetine treatment. This was possibly because of the very low expression of cGMP‐generating machinery in these cells. Therefore, we did not include the data showing this.

Next, we measured the effects of paclitaxel and vinpocetine on microtubule polymer mass. Paclitaxel alone significantly increased microtubule polymer mass in T50RN cells (Figure 5E, T), and vinpocetine alone slightly but significantly increased the microtubule polymer mass (Figure 5E, V), suggesting that vinpocetine might also stabilize tubulin polymers. Importantly, cotreatment of vinpocetine with paclitaxel markedly enhanced the paclitaxel‐induced microtubule polymer‐stabilizing effect (Figure 5E, T+ V). Tubulin acetylation is a critical post‐translational modification that regulates microtubule stability and dynamics (Janke and Magiera 2020). Our results showed that cotreatment of vinpocetine with paclitaxel enhanced the level of paclitaxel‐induced acetylated tubulin accumulation (Figure 5F), confirming that vinpocetine enhances the microtubule stabilizing effects of paclitaxel. Upon entering the cells, BAPTA‐AM is hydrolyzed to BAPTA, a potent cytosolic Ca^2+^ chelator, which noticeably mitigated the vinpocetine‐induced enhancement of tubulin acetylation in paclitaxel‐treated cells (Figure 5G). Up to this point, our results suggested that vinpocetine might enhance paclitaxel‐induced spindle abnormalities and cytotoxicity by increasing the microtubule polymer‐stabilizing effects of paclitaxel partially by disrupting PDE1C activity. An increase in the levels of Ca^2+^ and cAMP might be responsible for this enhancement effect.

### 3.5 Vinpocetine Increases TMRE Fluorescence Intensities and Induces ROS

**Figure 6:**
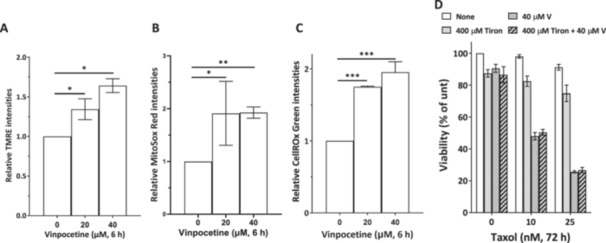
Vinpocetine induces ROS in T50RN cells. T50RN cells were treated with vinpocetine for 6 h at the indicated concentrations and then subjected to flow cytometry analysis of mitochondrial membrane potential (TMRE, A), mitochondrial superoxide (MitoSox, B), and cellular peroxides (CellRox, C). Significant differences in the data were assessed by Student's *t* test. *p < 0.05, **p < 0.01, ***p < 0.001 comparing between indicated groups. (D) Tiron did not reverse vinpocetine enhancement of paclitaxel sensitivity in T50RN cells. T50RN cells were treated as indicated for 72 h and then subjected to trypan blue exclusion viability assay. Data are shown as mean ± SD from three independent experiments.

Since our results suggested that vinpocetine might have targets other than PDE1 and it is known that mitochondria and ROS generation play critical roles in modulating microtubule dynamics and apoptosis induction (Bock and Tait 2020; Ong et al. 2020; Sinha et al. 2013), we next examined the effects of vinpocetine on mitochondrial function and ROS induction in T50RN cells. Mitochondrial function was assessed by detecting mitochondrial membrane potential with TMRE dye. ROS levels were measured by detecting fluorescence signals in cells stained with CellROX Green (which detects ROS) or MitoSox Red (which detects mitochondrial superoxides) by flow cytometry. The results showed that treatment of T50RN cells with vinpocetine for 6 h increased TMRE fluorescence intensities (Figure 6A) and significantly elevated mitochondrial superoxide (Figure 6B) and intracellular ROS (Figure 6C) levels. Thus, vinpocetine may induce mitochondrial hyperpolarization and stimulate ROS generation after 6 h of treatment in T50RN cells. The timing of these effects suggested that before enhancing the anti‐mitotic effects of paclitaxel (24 h), vinpocetine might induce mitochondria hyperpolarization and ROS generation (6 h) in T50RN cells. Nevertheless, simultaneous treatment of vinpocetine and the superoxide anion scavenger tiron did not affect vinpocetine effects on paclitaxel cytotoxicity in T50RN cells (Figure 6D).

### 3.6 Vinpocetine May Enhance T50RN Sensitivity to Paclitaxel by Disrupting P‐gp Function

Vinpocetine has been demonstrated to inhibit P‐gp/MDR1 efflux activity (Manda et al. 2015), which often plays a critical role in paclitaxel resistance (Murray et al. 2012). We thus examined whether vinpocetine could disrupt the activities of P‐gp in T50RN cells by performing rhodamine 123 accumulation and efflux assays. The results in Figure 3A,B show that expression levels of the P‐gp encoding gene *ABCB1* and P‐gp/MDR1 were significantly increased in T50RN cells compared to MDA‐MB‐231 cells. In addition, T50RN cells accumulated significantly less rhodamine than the parental MDA‐MD‐231 cells (Figure 3C). When rhodamine‐loaded cells were maintained in rhodamine‐free medium, the intracellular fluorescence intensity of T50RN cells decreased to 25% of the initial level within 0.5 h and kept decreasing thereafter; however, the intracellular fluorescence level in MDA‐MB‐231 cells was largely unchanged (Figure 3D). These results indicate that T50RN cells have higher (rhodamine 123) efflux activity than MDA‐MB‐231 cells. Zosuquidar (Zo) is a small molecule that binds with high affinity to P‐gp and inhibits P‐gp‐mediated multidrug resistance activity (Callies et al. 2003). Treatment with Zo had no effect on MDA‐MB‐231 cells, but it enhanced rhodamine 123 accumulation in T50RN cells, bringing it to a similar level as in MDA‐MB‐231 cells (Figure 3C). Vinpocetine treatment also enhanced rhodamine 123 accumulation (Figure 3C) and prevent efflux of preloaded rhodamine 123 (Figure 3D) in T50RN cells. However, vinpocetine had no noticeable effect on P‐gp/MDR1 protein expression (Figure 3E), indicating altering MDR1 protein expression might not be the cause of vinpocetine inhibition of P‐gp activities. These results confirmed that P‐gp activity is elevated in T50RN cells and may lead to paclitaxel resistance in T50RN cells. Furthermore, vinpocetine appears to disrupt P‐gp activity, leading to increased paclitaxel retention and enhanced cytotoxicity in T50RN cells. Finally, we examined whether inhibiting P‐gp in T50RN would alter the effects of vinpocetine on paclitaxel resistance. We found that Zo treatment could synergistically enhance paclitaxel cytotoxicity in T50RN cells as the CI of combined treatments are smaller than 1 (Figure 3F, red numbers) and the IC_50_ of paclitaxel decreased to 14.9 nM (Figure 1C). Furthermore, simultaneous addition of Zo and vinpocetine to paclitaxel‐treated T50RN cells slightly enhance the effects of vinpocetine on paclitaxel cytotoxicity in T50RN cells (Figure 3G). Thus, vinpocetine might act mainly by inhibiting P‐gp and PDE1C to enhance paclitaxel sensitivity.

## 4 Discussion

Vinpocetine is a widely marketed dietary supplement that is well tolerated for long‐term usage. Recent reports have suggested the supplement may be useful for treating or preventing cerebrovascular disorders (Patyar et al. 2011), cognitive dysfunction (Szatmari and Whitehouse 2003) or memory disorders (Ahmad et al. 2022). Vinpocetine has also been used in cancer patients receiving brain irradiation to reduce cognitive impairments (P. Zhang et al. 2021). An expected physiological effect of vinpocetine is vasodilatation, as it can inhibit PDE1 and increase cAMP and cGMP levels (Morgado et al. 2012). The molecule may also inhibit the release of pro‐inflammatory cytokines by suppressing the IKK/NF‐κB/activator protein‐1 pathway (Jeon et al. 2010). The neurological effects of vinpocetine may be due to inhibition of voltage‐gated sodium channels, reduction of neuronal calcium influx, and/or antioxidant effects (Y. S. Zhang et al. 2018). These wide‐ranging molecular targets and modes of action may jointly contribute to its effects in individuals. In this study, we found that vinpocetine can enhance paclitaxel cytotoxicity in the paclitaxel‐resistant T50RN TNBC cell line but not parental paclitaxel‐sensitive MDA‐MB‐231 cells. Our results show that vinpocetine most likely acts to enhance the cytotoxic and anti‐mitotic effects of paclitaxel in T50RN cells by inhibiting PDE1 and P‐gp activities. Our in vitro studies require further validation in additional paclitaxel‐resistant cell lines and in mouse xenograft models. This will allow us to determine whether vinpocetine overcomes paclitaxel resistance, paving the way for its future application.

The most well‐known action of vinpocetine is as a PDE1 inhibitor. PDE1 catalyzes both cAMP and cGMP degradation, so inhibition of PDE1 by vinpocetine would lead to accumulation of cAMP and cGMP. The accumulated cAMP and cGMP could possibly activate downstream kinase protein kinase A and protein kinase G, which then may trigger a series of Ca^2+^ signaling and related events. Importantly, regulation of microtubule dynamics is a major downstream effect of Ca^2+^ signaling (Akhmanova and Steinmetz 2015; Gudimchuk and McIntosh 2021). Our results showed that in addition to increasing the intracellular Ca^2+^ and cAMP levels in treated T50RN cells, vinpocetine could also induce a slight increase in microtubule polymer mass in T50RN cells. These results would be in line with vinpocetine‐induced PDE1 inhibition effects, as inhibition of PDE1 would be expected to increase intracellular Ca^2+^ and further enhance microtubule stabilization. Paclitaxel is a microtubule‐stabilizing drug that increases microtubule polymer mass. Our results showed that vinpocetine also increased cAMP levels and, concomitantly, further increased microtubule polymer mass in paclitaxel‐treated T50RN cells, suggesting that vinpocetine can enhance the anti‐mitotic effects of paclitaxel. However, our results also showed that depletion of PDE1C with specific shRNAs only moderately increased T50RN cell sensitivity to paclitaxel. Moreover, treatment of vinpocetine to PDE1C‐depleted T50RN cells further enhanced paclitaxel cytotoxicity. These results suggest that in addition to PDE1, vinpocetine may target other molecules to enhance paclitaxel cytotoxicity.

Vinpocetine is known to modulate antioxidant systems, and our results showed that vinpocetine dose‐dependently increased TMRE fluorescence. This result may indicate that the electron transport chain was hyper‐functional in vinpocetine‐treated cells, creating a strong proton gradient and thereby increasing ΔΦm and TMRE fluorescence intensity. It has been reported that vinpocetine could potentiate radiotherapy effects by increasing tumor *p*O2 and tumor oxygenation (Amano et al. 2005), suggesting vinpocetine might enhance ROS generation in multiple ways. It has also been shown that the antioxidant effects of vinpocetine occur mainly at low to moderate doses, while higher concentrations of vinpocetine lead to oxidative stress (Ansari et al. 2019). Our results would be consistent with vinpocetine enhancement of mitochondrial respiration, which may lead to increased mitochondria superoxide release and higher cellular ROS. However, tiron, a well‐known superoxide scavenger, did not obviously impact the paclitaxel‐enhancing effects of vinpocetine. This result indicated that superoxide generated by vinpocetine might not directly interact with tubulin or interfere with microtubule assembly to enhance paclitaxel cytotoxicity. Thus, we conclude that ROS generation is unlikely to play a critical role in the enhancement of paclitaxel‐mediated cytotoxicity by vinpocetine. Instead, this suggests the importance of vinpocetine's enhancing impact on paclitaxel's microtubule‐stabilizing effect.

Multidrug resistance is a major challenge in cancer chemotherapy. Our current results showed that T50RN cells accumulated much less rhodamine 123 than parental MDA‐MB‐231 cells, suggesting that the T50RN cells may have acquired drug efflux activities. The low accumulation of rhodamine 123 in T50RN cells was likely due to upregulated P‐gp, as the effect could be reversed by treatment with a specific P‐gp inhibitor. Vinpocetine not only enhanced rhodamine 123 accumulation in T50RN cells, but it also considerably inhibited efflux of rhodamine 123. Therefore, we concluded that vinpocetine may have anti‐P‐gp activity. This activity would explain how simultaneous treatment of vinpocetine and paclitaxel enhances paclitaxel cytotoxicity, as preventing paclitaxel efflux from T50RN cells would lead to its accumulation and increase cytotoxicity. In addition, inhibition of PDE1C by vinpocetine could further augment paclitaxel's anti‐mitotic effects. Thus, vinpocetine appears to enhance paclitaxel's effectiveness through a dual mechanism: it not only inhibited P‐gp to elevate the concentration of paclitaxel inside the cells but its inhibition of PDE1C might also augment paclitaxel's anti‐mitotic action, thereby making the mitotic machinery more susceptible. Furthermore, simultaneous treatment of vinpocetine with Zo and paclitaxel further enhanced T50RN cell sensitivity to paclitaxel, indicating that the anti‐P‐gp and PDE1 inhibition effects of vinpocetine may play a pivotal role in the restoration of paclitaxel sensitivity in T50RN cells.

## 5 Conclusions

It has been shown that vinpocetine acts on Akt/STAT3 and likely on IKK/NF‐κB signaling pathways to inhibit MDA‐MB‐231 cell proliferation (Huang et al. 2012). However, the vinpocetine concentrations we used in this study did not cause cytotoxicity in MDA‐MB‐231 or T50RN cells. Instead, we found that vinpocetine only enhanced paclitaxel sensitivity in T50RN cells. Since the *PDE1C* and *ABCB1* genes are both significantly upregulated in T50RN cells, it was highly likely that vinpocetine may disrupt PDE1 and/or P‐gp activities to enhance T50RN cell sensitivity to paclitaxel. The concentration of vinpocetine used in this study is relatively higher than the concentration typically used by the general population as a dietary supplement or in clinics. It has been shown that, after oral administration of 20 mg, the vinpocetine plasma concentration reaches 62 or 200 ng/mL (about 0.2 μM or 0.6 μM) (Vereczkey et al. 1979; Vlase et al. 2005). For cognitive enhancement and cerebrovascular disorders, 60 mg per day has been suggested. These reports suggest that plasma concentrations of vinpocetine could gradually reach several μM. Vinpocetine is a widely consumed dietary supplement, and its source and purity may vary widely. Therefore, it should be used with caution. Our results also suggest that users of vinpocetine should be cautious of its anti‐P‐gp activity, which may affect the efflux and change pharmacokinetic profiles of many drugs. In addition, Nonetheless, our results suggest that vinpocetine may be a potential agent for overcoming paclitaxel resistance in TNBC cells in culture.

## Author Contributions

H.H.K. performed the establishment of T50RN cells, assays for cytotoxicity and mitotic spindle. C.W.H. performed the analysis of Ca^2+^, ROS, cell cycle, and apoptosis. W.R.C. performed the analysis of cytotoxicity, mitotic spindle, and microtubule polymer mass. L.H.Y. contributed to the conception and design of the work, interpreted the data, and drafted the manuscript.

## Ethics Statement

The authors have nothing to report.

## Consent

The authors have nothing to report.

## Conflicts of Interest

The authors declare no conflicts of interest.

## Data Availability

The data that support the findings of this study are available from the corresponding author upon reasonable request.
